# Two novel human anti-ErbB2 immunoagents are active on trastuzumab-resistant tumours

**DOI:** 10.1038/sj.bjc.6605499

**Published:** 2010-01-05

**Authors:** T Gelardi, V Damiano, R Rosa, R Bianco, R Cozzolino, G Tortora, P Laccetti, G D'Alessio, C De Lorenzo

**Affiliations:** 1 Dipartimento di Endocrinologia e Oncologia Molecolare e Clinica, Università di Napoli Federico II, via Pansini 5, Napoli 80131, Italy; 2 Dipartimento di Biologia Strutturale e Funzionale, Università di Napoli Federico II, via Cinthia, Napoli 80126, Italy

**Keywords:** ErbB2/Her2, immunotherapy, ImmunoRNase, trastuzumab, resistance

## Abstract

**Background:**

Overexpression of ErbB2 receptor in breast cancer is associated with disease progression and poor prognosis. Trastuzumab, the only humanised anti-ErbB2 antibody currently used in breast cancer, has proven to be effective; however, a relevant problem for clinical practice is that a high fraction of breast cancer patients shows primary or acquired resistance to trastuzumab treatment.

**Methods:**

We tested on trastuzumab-resistant cells two novel human anti-tumour immunoconjugates engineered in our laboratory by fusion of a human anti-ErbB2 scFv, termed Erbicin, with either a human RNase or the Fc region of a human IgG1. Both Erbicin-derived immunoagents (EDIAs) are selectively cytotoxic for ErbB2-positive cancer cells *in vitro* and *vivo*, target an ErbB2 epitope different from that recognised by trastuzumab and do not show cardiotoxic effects.

**Results:**

We report that EDIAs are active also on trastuzumab-resistant tumour cells both *in vitro* and *in vivo*, most likely because of the different epitope recognised, as EDIAs, unlike trastuzumab, were found to be able to inhibit the signalling pathway downstream of ErbB2.

**Conclusion:**

These results suggest that EDIAs are immunoagents that could not only fulfil the therapeutic need of patients ineligible to trastuzumab treatment due to cardiac dysfunction but also prove to be useful for breast cancer patients unresponsive to trastuzumab treatment.

Breast cancer is a disease with more than one million new diagnoses worldwide each year and with a high risk of metastases. Several studies have shown that up to 25% of breast cancers overexpress a member of the HER/ErbB transmembrane receptor tyrosine kinase family, ErbB2, associated with poor prognosis and with a more aggressive clinical behaviour (Baselga and Albanell, 2001). Overexpression of ErbB2 leads to activation of the ErbB2-mediated signalling pathways, including the mitogen-activated protein kinase (MAPK) and phosphatidylinositol-3-kinase pathways. The first ErbB2-targeted therapy approved by the United States Food and Drug Administration is trastuzumab, a humanised monoclonal antibody that binds to the extracellular domain of HER2. Trastuzumab development has led to a significant improvement in the outcomes of patients with ErbB2-positive breast cancer. It has been reported that trastuzumab induces internalisation and downregulation of ErbB2 (Sliwkowski *et al*, 1999), cell cycle arrest (Baselga and Albanell, 2001; Kim *et al*, 2003), inhibition of angiogenesis (Izumi *et al*, 2002) and induction of antibody-dependent cell-mediated cytotoxicity (ADCC) (Clynes *et al*, 2000). Several studies showed that the use of trastuzumab alone in first-line metastatic breast cancer resulted in a median overall response rate of 19–26% (Vogel *et al*, 2002). Moreover, in the first-line setting, the use of trastuzumab in combination with chemotherapy results in a longer time to disease progression, a higher rate of objective response, a longer duration of response and a 20% reduction in the risk of death (Slamon *et al*, 2001).

However, many patients with HER2-positive metastatic breast cancer do not respond to trastuzumab, or eventually become resistant to it. For these patients, the prognosis is poor and the disease progresses more aggressively. Resistance to trastuzumab is often associated with an increased expression of the membrane-associated glycoprotein mucin-4 (MUC4), which binds and sterically hinders ErbB2 from binding to trastuzumab. Furthermore, MUC4 can also activate the receptor through its epidermal growth factor (EGF)-like domain (Nagy *et al*, 2005).

The JIMT-1 cell line was isolated from a pleural effusion in a patient with metastatic ErbB2-driven breast disease who was clinically resistant to trastuzumab therapy. This cell line shows *her2/ErbB2* gene amplification and an elevated MUC4 expression level, which masks the trastuzumab-binding epitopes of ErbB2 (Tanner *et al*, 2004). The KPL-4 cell line was isolated from the malignant pleural effusion of a breast cancer patient with an inflammatory skin metastasis; in female athymic nude mice, KPL-4 cells are mostly resistant to trastuzumab (Kurebayashi *et al*, 1999).

Two novel human anti-ErbB2 immunoconjugates have been engineered in our laboratory by fusion of a human scFv (De Lorenzo *et al*, 2002), termed Erbicin, with either a human RNase or the Fc region of a human IgG1. The former is called Erb-hRNase, the latter human anti-ErbB2-compact Antibody (Erb-hcAb). Both immunoagents are selectively cytotoxic for ErbB2-positive cancer cells *in vitro* and *in vivo* (De Lorenzo *et al*, 2004a,2004b, 2005).

These Erbicin-derived immunoagents (EDIAs) target an ErbB2 epitope on breast cancer cells different from that of trastuzumab (De Lorenzo *et al*, 2007a). Previous reports have suggested that the epitope recognised by trastuzumab may be masked in trastuzumab-resistant tumour cells (Nagy *et al*, 2005; Valabrega *et al*, 2007), given that they could still be sensitive to treatment with antibodies recognising different epitopes. Thus, the use of EDIAs, which recognise a different site on ErbB2, could prove to be effective for treatment of breast cancer that cannot be cured with trastuzumab.

In this study, we evaluated whether our agents are effective also in cancer cell lines with spontaneous or acquired resistance to trastuzumab, *in vitro* and *in vivo*.

## Materials and methods

### Cell cultures

The KPL-4 cell line was isolated from the malignant pleural effusion of a breast cancer patient with an inflammatory skin metastasis; in female athymic nude mice, KPL-4 cells are mostly resistant to trastuzumab (Kurebayashi *et al*, 1999). The JIMT-1 cell line was established from a pleural metastasis of a 62-year-old patient with breast cancer who was clinically resistant to trastuzumab, and forms trastuzumab-resistant xenografts in nude mice (Tanner *et al*, 2004). KPL-4, JIMT-1 and MDA-MB 361 breast cancer cells were maintained in RPMI medium supplemented with 10% heat-inactivated foetal bovine serum, 20 mM HEPES, pH 7.4, penicillin (100 UI ml^−1^), streptomycin (100 *μ*g ml^−1^) and 4 mM glutamine (ICN, Irvine, UK) in a humidified atmosphere of 95% air and 5% CO_2_ at 37°C.

### Compounds

The antibodies used were trastuzumab (Genentech, South San Francisco, CA, USA), horseradish peroxidase-conjugated goat anti-human affinity-isolated IgG1 (Fc-specific; Sigma, St Louis, MO, USA), Erb-hcAb and Erb-hRNase. These latter were prepared as described previously (De Lorenzo *et al*, 2004a,2004b).

### Western blot analysis

Total cell lysates were obtained from cellular lysates or homogenised tumour specimens removed on day 25. Protein extracts were resolved by 4–15% SDS–polyacrylamide gel electrophoresis and probed with anti-human, monoclonal pEGFR, polyclonal EGFR, polyclonal pHER2, monoclonal HER2, monoclonal pMAPK and monoclonal MAPK, monoclonal vascular endothelial growth factor (Santa Cruz Biotechnology, Santa Cruz, CA, USA), monoclonal pAkt, polyclonal Akt (Cell Signaling Technologies, Beverly, MA, USA) and monoclonal actin (Sigma-Aldrich, Milan, Italy). Immunoreactive proteins were visualised by enhanced chemiluminescence (Pierce, Rockford, IL, USA), as previously described (Ciardiello *et al*, 2004).

### Growth in soft agar

On day 0, cells were suspended in 0.3% Difco Noble agar (Difco, Detroit, MI, USA) supplemented with complete culture medium, layered over 0.5 ml of 0.8% agar medium base and treated with different nM concentrations of Erb-hRNase and Erb-hcAb alone. After 10–14 days, cells were stained with nitro blue tetrazolium (Sigma Chemical Co., Milan, Italy) and colonies >0.05 mm were counted (Ciardiello *et al*, 2004). Typically, s.d. values were below 10%.

### *In vitro* assay of drug sensitivity in the absence of serum

To test the effects of EDIA on the growth of JIMT-1 and KPL-4 cell lines, cells were plated at a density of 5000 cells per well in 96-well, flat-bottomed, tissue culture plates deprived of serum for the following 24 h and then treated with immunoagents (50–200 nM) for 72 h, as previously reported (Tanner *et al*, 2004). Cell counts were determined in triplicate using the trypan blue exclusion test and survival was expressed as percentage of viable treated cells with respect to control untreated cultures. Typically, s.d. values were below 10%.

### Xenografts in nude mice

Balb/cAnNCrlBR athymic (nu/nu) mice (5 weeks old) (Charles River Laboratories, Milan, Italy) were maintained in accordance with the institutional guidelines of the University of Naples Animal Care Committee and in accordance with the Declaration of Helsinki and the Italian regulations for the welfare of animals used in studies of experimental neoplasia. The study was approved by the School of Medicine Institutional Committee on animal care.

KPL-4 or JIMT-1 human breast cancer cells (10^7^ cells per mice) were resuspended in 200 *μ*l of Matrigel (Collaborative Biomedical Products, Bedford, MA, USA) and injected subcutaneously into mice. After 7 days, tumours were detected and groups of 10 mice were randomised to receive the following treatments: intraperitoneal ImmunoRNase, dissolved in phosphate-buffered saline, was administered at doses of 1.5 mg kg^−1^ of body weight for six times at 72-h intervals; and intraperitoneal trastuzumab, 3.75 mg kg^−1^ and Erb-hcAb 2.5 mg kg^−1^ were administered twice a week for 3 weeks. Tumour volume was measured using the formula *π*/6 × larger diameter × (smaller diameter)^2^, as previously reported (Ciardiello *et al*, 1996). Two mice from each group were killed on day 25 to perform biochemical analysis.

## Results

### Effects of EDIA on human breast cancer cells sensitive or resistant to trastuzumab

**Figure 1:**
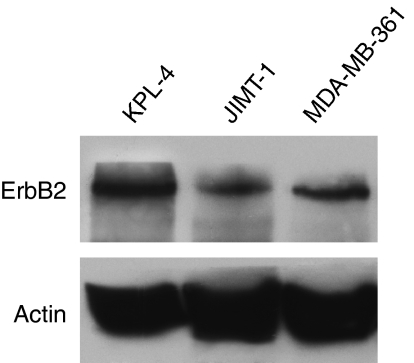
ErbB2 expression on human breast cancer cell lines. Western blot analysis of ErbB2 protein expression on total cell lysates from KPL-4, JIMT-1 and MDA-MB-361cells.

We measured the expression of ErbB2 on breast cancer cell lines JIMT-1 and KPL-4 (Kurebayashi *et al*, 1999; Tanner *et al*, 2004), directly derived from patients with aggressive trastuzumab-resistant tumours, and on MDA-MB-361 cells that are sensitive to trastuzumab. A western blot analysis with an anti-ErbB2 monoclonal antibody was performed on lysates from the aforementioned cancer cell lines. As shown in Figure 1, all cancer cell lines express, at different levels, the ErbB2 receptor.

**Figure 2:**
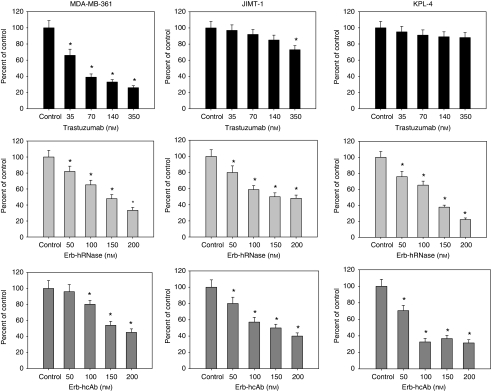
Effects of trastuzumab, ERB-hRNase and ERB-hcAb on the soft agar growth of MDA-MB-361, JIMT-1 and KPL-4 cells. Cells were treated with the indicated concentrations of trastuzumab, Erb-hRNase and Erb-hcAb each day for three consecutive days. Colonies were counted after 10–14 days. Results for each treatment are presented relative to untreated control cells. ^*^Two-sided *P*<0.0001 *vs* control and *vs* negative control. Bars, s.d.

To test the anti-proliferative effects of anti-ErbB2 immunoagents on cancer cell growth, we treated these cells in soft agar with different doses of immunoagents. As shown in Figure 2, both Erb-hRNase and Erb-hcAb caused a dose-dependent inhibition of colony formation in all cancer cells tested, proving to be effective also in trastuzumab-resistant JIMT-1 and KPL-4 cells, with an IC_50_ (concentration producing an inhibition of 50%) value of ∼50 and 100 nM, respectively. No growth inhibition or very limited inhibition (<20% for JIMT1 cells) was observed, as expected (Tanner *et al*, 2004), when trastuzumab was tested in parallel experiments carried out under identical conditions.

**Figure 3:**
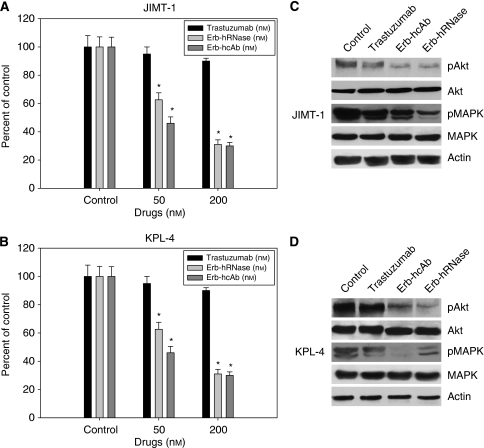
Effects of trastuzumab, Erb-hRNase and Erb-hcAb on survival (**A** and **B**) and signal transduction (**C** and **D**) of JIMT-1 and KPL-4 cells. (**A** and **B**) 5000 cells per well were plated in 96-well tissue culture plates for 48 h in the presence of 7.5% serum, then deprived of serum for the following 24 h and finally treated with trastuzumab, Erb-hRNase or Erb-hcAb (50–200 nM) in 0.1% serum for 72 h. Cell counts were performed using trypan blue. Results for each treatment are presented relative to that of untreated control cells. ^*^Two-sided *P*<0.0001 *vs* control and *vs* negative control. Bars, s.d. (**C** and **D**) Western blot analysis of protein expression in JIMT-1 and KPL-4 cells treated for 24 h with trastuzumab (150 nM), Erb-hRNase (50 nM) and Erb-hcAb (100 nM) before protein extraction. For each immunoagent, the minimum concentration that gave clear effects was chosen.

We also performed a cell-survival assay and observed that anti-ErbB2 immunoagents caused a potent effect on the survival of JIMT-1 (Figure 3A) and KPL-4 (Figure 3B) cells. As expected, trastuzumab was ineffective on cell survival, whereas Erb-hRNase and Erb-hcAb induced a 50% cell-survival inhibition in JIMT-1 and KPL-4 cells at a dose of 50 nM for both immunoagents.

We evaluated by western blot analysis the effects of treatment with Erb-hRNase and Erb-hcAb on the expression of proteins involved in the ErbB2 pathway in JIMT-1 and KPL-4 cells. As shown in Figure 3C, Erb-hcAb and Erb-hRNase inhibited the phosphorylation of MAPK and Akt more effectively than did trastuzumab, thus suggesting that, unlike trastuzumab, EDIAs retain their ability to inhibit the ErbB2 survival pathway in trastuzumab-resistant cells.

### *In vivo* anti-tumour effects of EDIA on trastuzumab-resistant breast cancer cells grown in nude mice

**Figure 4:**
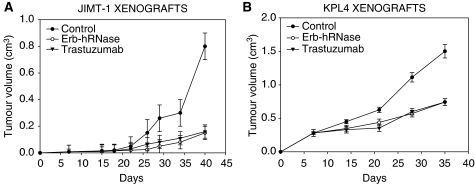
Effect of Erb-hRNase and trastuzumab on tumour growth of JIMT-1 (**A**) and KPL-4 (**B**) xenografts. On day 7, when tumours were clearly detectable, mice were randomised (10 per group) to receive the following treatments: intraperitoneal (i.p.) trastuzumab (3.75 mg kg^−1^ twice a week for 3 weeks) or i.p. ERB-hRNase (1.5 mg kg^−1^, at 72-h intervals for 3 weeks). Control animals were treated with sterile phosphate-buffered saline solution. Student's *t*-test was used to compare tumour sizes among different treatment groups. Bars, s.d.; *P*-value 0.027.

In a first study, we xenografted BalbC nude mice with JIMT-1 and KPL-4 tumours and treated the animals with trastuzumab and Erb-hRNase (Figure 4). After 7 days, when tumours were clearly detectable, mice were treated. Treatment with trastuzumab and Erb-hRNase showed a similar effect on tumour growth, producing about 70 and 50% tumour growth inhibition in JIMT-1 and KPL-4 tumour models, respectively, at the end of the experiment (Figures 4A and B).

In two different experiments, BalbC nude mice were xenografted with the same tumour cells and treated with Erb-hcAb. In the first experiment, when tumours were detectable, mice were treated with 3.3 mg kg^−1^ of Erb-hcAb once a week by systemic administrations. To directly compare the anti-tumour efficacy of this novel immunoagent with that of trastuzumab, in parallel experiments, the effects of equimolar doses (5 mg kg^−1^) of trastuzumab were tested on the same experimental models, as previously reported (Tanner *et al*, 2004). Tumour volume was measured weekly until the end of the experiments. We found that Erb-hcAb inhibited the growth of tumours during the course of treatment, and its effects were significantly more potent than that of trastuzumab (75 *vs* 30% growth inhibition; *P*-value 0.033).

**Figure 5:**
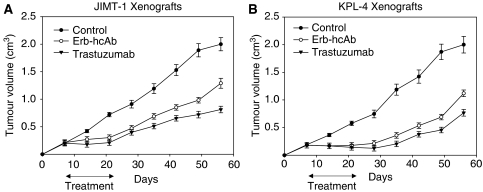
Effect of Erb-hcAb and trastuzumab on tumour growth of JIMT-1 (**A**) and KPL-4 (**B**) xenografts. On day 7, when tumours were clearly detectable, mice were randomised (10 per group) to receive the following treatments: intraperitoneal (i.p.) trastuzumab (3.75 mg kg^−1^, twice a week for 3 weeks) or i.p. Erb-hcAb (2.5 mg kg^−1^, twice a week for 3 weeks). Control animals were treated with sterile phosphate-buffered saline solution. Student's *t*-test was used to compare tumour sizes among different treatment groups. Bars, s.d.

In the second experiment, treatment was performed on larger-sized tumours with two doses at 72-h intervals of trastuzumab (3.75 mg kg^−1^) or Erb-hcAb (2.5 mg kg^−1^). On day 56, 8 weeks after tumour injection, all untreated mice bearing JIMT-1 and KPL-4 tumours reached the maximum allowed tumour size of about 2 cm^3^. On day 49, 3 weeks after treatment withdrawal, Erb-hcAb produced about 50 and 65% tumour growth inhibition in JIMT-1 and KPL-4 tumour models, respectively (Figures 5A and B), whereas trastuzumab produced about 75 and 65% tumour growth inhibition in JIMT-1 and KPL-4 tumour models, respectively (Figures 5A and B). These data on trastuzumab activity are consistent with those of a previous study showing that trastuzumab is able to inhibit the outgrowth of macroscopically detectable JIMT-1 xenografted tumours, despite the *in vitro* intrinsic resistance of JIMT1 cells to trastuzumab. The different response of these cells *in vitro* and *in vivo* may not be surprising, as it can be attributed to the well-documented ADCC of this agent (Barok *et al*, 2007, 2008).

These findings reflect the central role of the immune system in mediating the effects of trastuzumab *in vivo*. If the clinical effects of trastuzumab on tumour masses are mainly derived from ADCC, which could lose its efficacy as the tumour grows, it is possible that clinical benefits may decrease during extended follow-up and would thus benefit from combinatorial use of Erb-hcAb, on the basis of a different mechanism of action.

### EDIAs inhibit the expression of signalling proteins in KPL-4 and JIMT-1 xenografts

**Figure 6:**
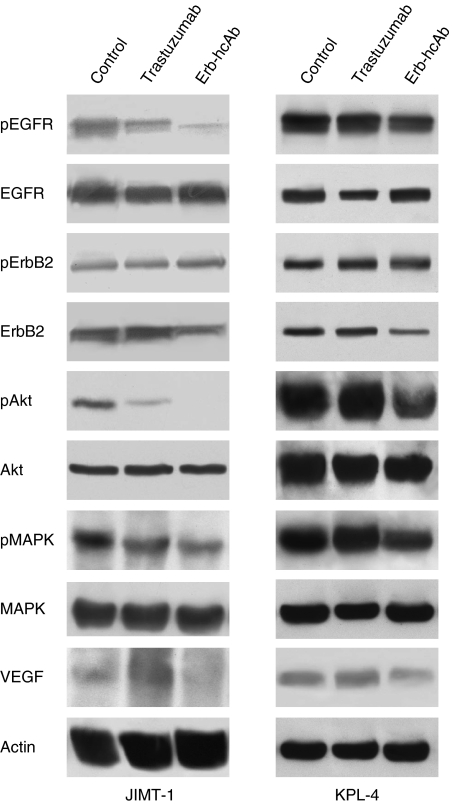
Effect of Erb-hcAb and trastuzumab on signal transduction in JIMT-1 and KPL-4 tumour xenografts. Western blotting was performed on total lysates from tumour specimens of two mice killed on day 25 and treated as in Figure 5.

**Figure 7:**
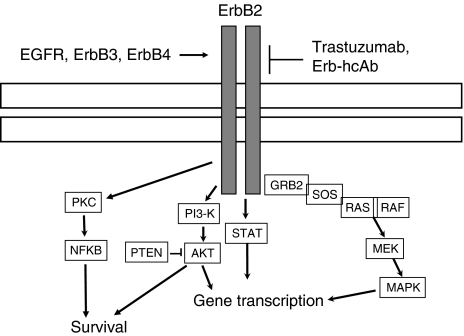
Downstream effectors of the ErbB receptor signalling pathway. AKT, protein kinase B; MAPK, mitogen-activated protein kinase; MEK, MAPK/extracellular signal-related kinase; NF-*κ*B, nuclear factor *κ*B; PKC, protein kinase C; PI3K, phosphatidylinositol 3-kinase; PTEN, phosphatidylinositol-3,4,5-trisphosphate-3-phosphatase; STAT, signal transducer and activator of transcription.

We analysed the effects of treatment on the expression of several proteins having a critical role in cancer cell proliferation and angiogenesis. Western blotting analysis was performed on cell lysates from tumours removed at the end of the third week of treatment, on day 25. As shown in Figure 6, both in JIMT-1 and KPL-4 tumour xenografts, Erb-hcAb inhibited the phosphorylation/activation of EGFR, Akt and MAPK, which are involved in ErbB2 signalling cascade as shown in Figure 7. Conversely, trastuzumab did not affect at all, and only slightly reduced the level of these proteins in KPL-4 and JIMT-1 cells, respectively. Furthermore, Erb-hcAb reduced the expressions of ErbB2 and vascular endothelial growth factor in treated cells as compared with untreated tumours. On the contrary, trastuzumab did not affect the level of ErbB2, differently from previous reports on sensitive mammary tumours (Petit *et al*, 1997; Sliwkowski *et al*, 1999).

## Discussion

Although trastuzumab has been successfully used in patients with ErbB2-overexpressing metastatic breast cancer, the objective response rate to trastuzumab monotherapy is 12–34% for a median duration of 9 months, when most patients become resistant to treatment (Cobleigh *et al*, 1999).

Primary or acquired resistance is such a frequent problem that it ultimately culminates in treatment failure. Thus, overcoming trastuzumab resistance represents an important challenge in the improvement of immunotherapy outcomes.

The mechanisms underlying the acquisition of trastuzumab resistance are not yet well defined, but pre-clinical studies have indicated several molecular mechanisms that could contribute to the development of trastuzumab resistance (Nahta and Esteva, 2006). Increased signalling through the PI3-K/Akt pathway could induce the activation of multiple receptor pathways that include ErbB-related receptors or non-ErbB receptors such as IGF1-R, which seems to be involved in a cross-talk with ErbB2 in resistant cells (Lu *et al*, 2001).

It has also been proposed that trastuzumab resistance may be associated with decreased p27(kip1) levels (Nahta *et al*, 2004), or with the loss of function of the tumour-suppressor PTEN gene, the negative regulator of Akt, which results in strengthened Akt signalling, which in turn leads to decreased sensitivity to trastuzumab (Fujita *et al*, 2006).

Other evidence suggests that ErbB2-positive cells may develop trastuzumab resistance by masking the epitope recognised by this antibody. Recent studies have shown that a decreased interaction between trastuzumab and ErbB2 is due to steric hindrance of ErbB2 by cell surface proteins such as MUC4. An example is the JIMT-1 cell line, established from the pleural effusion of a trastuzumab-resistant breast cancer patient, in which the membrane-proximal trastuzumab epitope is masked (Nagy *et al*, 2005).

The role of the epitope recognised by anti-ErbB2 antibodies is further highlighted by the preservation of the binding efficiency of an antibody such as 2C4, which recognises a membrane-distant epitope in the extracellular part of ErbB2 (Nagy *et al*, 2005).

Other results indicate that lack of accessibility of the epitope recognised by trastuzumab may limit the activity of this antibody *in vivo*: only about half of ErbB2 amplification cases confirmed by fluorescent *in situ* hybridisation (FISH) was found to be positive to trastuzumab staining. Interestingly, the efficacy of trastuzumab-based therapy was significantly higher in patients whose tumours stained positively for trastuzumab, despite all tumours being FISH positive (Bussolati *et al*, 2005). However, no cases of structural modifications or altered expression levels of ErbB2 in resistant cell lines have been reported.

On the basis of these considerations and the observations reported above, we assumed that cells that become resistant to trastuzumab could still be sensitive to treatment with antibodies recognising different epitopes or interfering with different intracellular signalling.

In this study, we found indeed that EDIAs are active on trastuzumab-resistant cells, inhibiting the expression of proteins involved in progression, survival and metastatic processes also. We observed that trastuzumab inhibited tumour growth *in vivo*, most likely because of ADCC, as previously reported (Barok *et al*, 2007), but we also showed that trastuzumab did not inhibit the expression of signalling protein, whereas Erb-hcAb and Erb-hRNase were able to inhibit not only tumour growth but also signal transducers. We believe that the sensitivity of trastuzumab-resistant cancer cells to EDIA is likely due to the different epitope recognised by EDIA.

Recent investigations on the mechanism of anti-tumour action of these anti-ErbB2 immunoagents have indicated that Erb-hcAb induces the homodimerisation of ErbB2, which leads to its downregulation and lysosomal degradation, and induces ADCC (De Lorenzo *et al*, 2005); the anti-tumour action of ERB-hRNase instead depends on its ribonuclease activity, which is exerted in the cytosol (De Lorenzo *et al*, 2007b). Thus, it may not be surprising that both immunoagents show similar cytotoxic effects, as Erb-hcAb lacks the toxic RNase payload of Erb-hRNase, but in turn the latter does not possess the Fc domain responsible for ADCC, and contains a monovalent scFv that could not significantly interfere with receptor dimerisation and degradation.

Moreover, a recent study showed that, unlike trastuzumab, EDIAs do not show cardiotoxicity (Riccio *et al*, 2009). In this study, Erb-hRNase and Erb-hcAb did not show toxic effects both *in vitro* on rat cardiomyocytes and *in vivo* on a mouse model, whereas trastuzumab was strongly toxic, thus suggesting that EDIA could be safely used for therapeutic applications.

Altogether, these results suggest that EDIA could fulfil the therapeutic need of patients ineligible for trastuzumab treatment because of cardiac dysfunction, and could prove to be effective for treatment of some breast cancer patients resistant to trastuzumab, providing a strong rationale for a clinical translation in breast cancer patients.
